# In vitro demetalation of central magnesium in various chlorophyll derivatives using Mg-dechelatase homolog from the chloroflexi *Anaerolineae*

**DOI:** 10.1007/s11120-024-01088-4

**Published:** 2024-03-26

**Authors:** Soma Sato, Mitsuaki Hirose, Ryouichi Tanaka, Hisashi Ito, Hitoshi Tamiaki

**Affiliations:** 1 Graduate School of Life Sciences, Ritsumeikan University, Kusatsu, Shiga 525-8577 Japan; 2 Graduate School of Environmental Science, Hokkaido University, N10 W5, Sapporo, 060-0810 Japan; 3 Institute of Low Temperature Science, Hokkaido University, N19 W8, Sapporo, 060-0819 Japan; 4 Department of Science and Technology, Seikei University, Tokyo, 180-8633 Japan

**Keywords:** Chlorophyll degradation, Stay-Green, Substitution effect, Substrate specificity

## Abstract

In the metabolic pathway of chlorophylls (Chls), an enzyme called STAY-GREEN or SGR catalyzes the removal of the central magnesium ion of Chls and their derivatives to their corresponding free bases, including pheophytins. The substrate specificity of SGR has been investigated through in vitro reactions using Chl-related molecules. However, information about the biochemical properties and reaction mechanisms of SGR and its substrate specificity remains elusive. In this study, we synthesized various Chl derivatives and investigated their in vitro dechelations using an SGR enzyme. Chl-*a* derivatives with the C3-vinyl group on the A-ring, which is commonly found as a substituent in natural substrates, and their analogs with ethyl, hydroxymethyl, formyl, and styryl groups at the C3-position were prepared as substrates. In vitro dechelatase reactions of these substrates were performed using an SGR enzyme derived from an *Anaerolineae* bacterium, allowing us to investigate their specificity. Reactivity was reduced for substrates with an electron-withdrawing formyl or sterically demanding styryl group at the C3-position. Furthermore, the Chl derivative with the C8-styryl group on the B-ring was less reactive for SGR dechelation than the C3-styryl substrate. These results indicate that the SGR enzyme recognizes substituents on the B-ring of substrates more than those on the A-ring.

**Supplementary Information:**

The online version contains supplementary material available at 10.1007/s11120-024-01088-4.

## Introduction

Deciduous plants change their leaf color from green to red or orange during the transition from summer to autumn. This phenomenon occurs because of chlorophyll (Chl) degradation, which is a process that serves as a protective mechanism in plants by preventing the generation of reactive oxygen species due to photoinhibition (Kräutler 2019). The first step of Chl degradation involves a reaction in which the central magnesium is detached, which is catalyzed by an enzyme known as Stay-Green (SGR, Shimoda et al. 2016). According to the literature, SGR is not exclusive to photosynthetic plants but is also found in non-photosynthetic bacteria. For example, the recombinant SGR from the Chloroflexi *Anaerolineae* SM23_63 exhibited high magnesium dechelation activity when it was overexpressed in *Escherichia coli* (Obata et al. 2019)*.* In this study, we further investigated its enzymatic properties to gain insights into its reaction mechanism. Hereafter, this enzyme is simply referred to “the SGR homolog”.

**Fig. 1:**
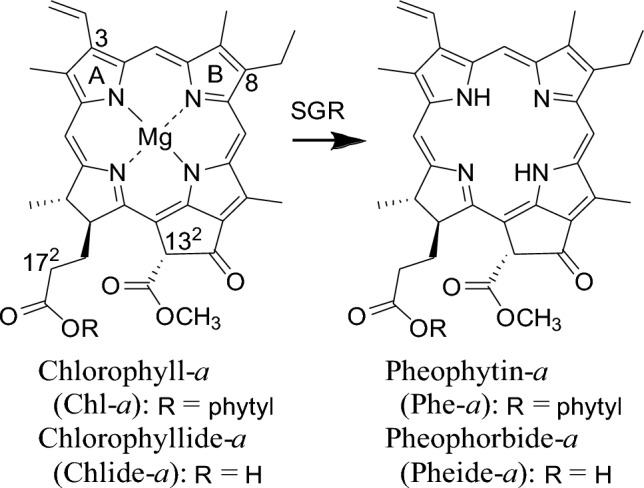
SGR enzyme reaction

**Fig. 2:**
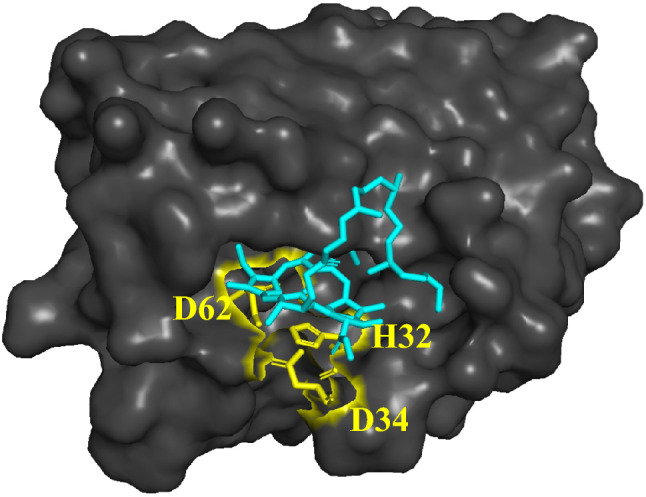
Docking simulation of AbSGR-h with Chl-*a*. Chl-*a*, AbSGR-h, and three catalytic amino acid residues are colored in blue, black, and yellow, respectively (Dey et al. 2022): PDB ID of 7Y5Y as AbSGR-h

The SGR homolog exhibits low substrate specificity. Chl-*a* and chlorophyllide-*a* (Chlide-*a*) as substrates for plant SGR are effective for in vitro reactions (Fig. 1), and protochlorophyllide-*a* and Chls-*b*/*c,* which cannot be used as substrates for plant SGR, are dechelated by the SGR homolog (Obata et al. 2019). Recently, the crystal structure of the SGR homolog has been determined (Dey et al. 2022), and its activity site has been proposed to be involved in the three amino acid residues of H32, D34, and D62 (Fig. 2). Based on its crystal structure and docking simulation with Chl-*a*, it has been proposed that D34 coordinates with the central Mg in Chl-*a* and destabilizes it, whereas H32 interacts with D62 to readily deprotonate D34. In addition, it is anticipated that the reaction mechanism involves electrostatic interactions at D62, which facilitates the deprotonation of H32 (Dey et al. 2021). Because the crystal structure of the enzyme–substrate complex remains unclear, the precise reaction mechanism, including the substrate recognition site, remains unclear.

**Fig. 3:**
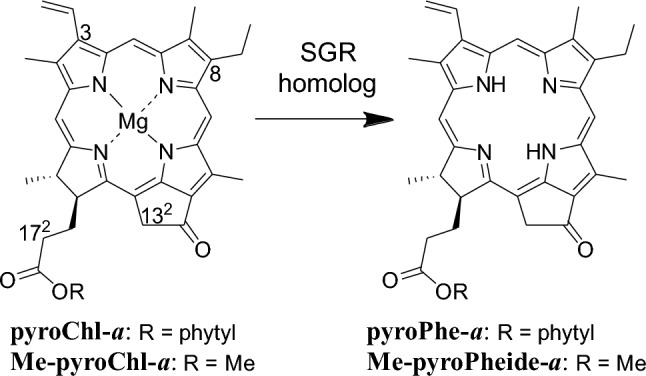
The present in vitro demetallation (dechelation) of Chl-*a* derivatives to the corresponding free bases by an SGR homolog

In this study, various Chl-*a* derivatives were synthesized and used as substrates to investigate the substrate specificity of the SGR homolog. Initially, pyrochlorophyll-*a* (**pyroChl-*a***) without the 13^2^-methoxycarbonyl group was synthesized as a substrate that is more chemically stable than Chl-*a* and its derivatives with the 13^2^-COOCH_3_ moiety, and the reactivity of the synthesized sample was compared with that of Chl-*a* (Fig. 3). Next, methyl pyrochlorophyllide-*a* (**Me-pyroChl-*a***) with the 17^2^-COOCH_3_ group was prepared and subjected to in vitro SGR reactions (Fig. 3). Furthermore, its C3/C8-substituted derivatives were produced, and their SGR reactivities were examined. Electron-withdrawing formyl and sterically bulky styryl groups suppressed the dechelations, and the steric enhancement of the C8-substituent on the B-ring reduced reactivity more than that at the C3-position on the A-ring. Based on the in vitro reactions, the SGR dechelation mechanism is discussed.

## Materials and methods

### General

One-dimensional proton nuclear magnetic resonance spectra of CDCl_3_ were recorded on a JEOL JNM‐ECA‐600 (600 MHz); residual CHCl_3_ (δ = 7.26 ppm) was used as an internal reference. High-performance liquid chromatography (HPLC) was performed using a Shimadzu Prominence liquid chromatography system with an SCL-10Avp system controller, an SPD-M20A photodiode array detector (300–800 nm), two LC-20AD pumps, a DGU-20A_3_ degasser, and a CTO-20AC column oven. Liquid chromatography–mass spectrometry (LC–MS) was performed using a Shimadzu LCMS-2010EV system, which was based on the atmospheric pressure chemical ionization mode (Hirose et al. 2022). For all LC(-MS) analyses, the column oven was set to 30 °C.

Flash column chromatography (FCC) and reversed-phase HPLC were performed using silica gels (Merck Kieselgel 60, 0.040–0.063 mm or Wakogel C-300) and a packed octadecylated silica gel column (Cosmosil 5C_18_-AR-II, Nacalai Tesque), respectively. The sample was dissolved in an HPLC eluent. The solution was filtered using a Cosmonice filter (0.45 μm pore size, Nacalai Tesque), and the filtrate was subjected to HPLC. Methanol and distilled water for HPLC solvents were purchased as HPLC grade from Nacalai Tesque. Acetone for HPLC grade was purchased from FUJIFILM Wako Pure Chemical.

### Preparation of recombinant AbSGR-h

The *Anaerolineae* bacterium SM23_63 SGR homolog (AbSGR-h) enzyme was prepared by homogenizing and extract of SM23_63 SGR expressed in BL21 *Escherichia coli* according to a previous procedure (Dey et al. 2021).

### Synthesis of substrates and authentic products

**Fig. 4:**
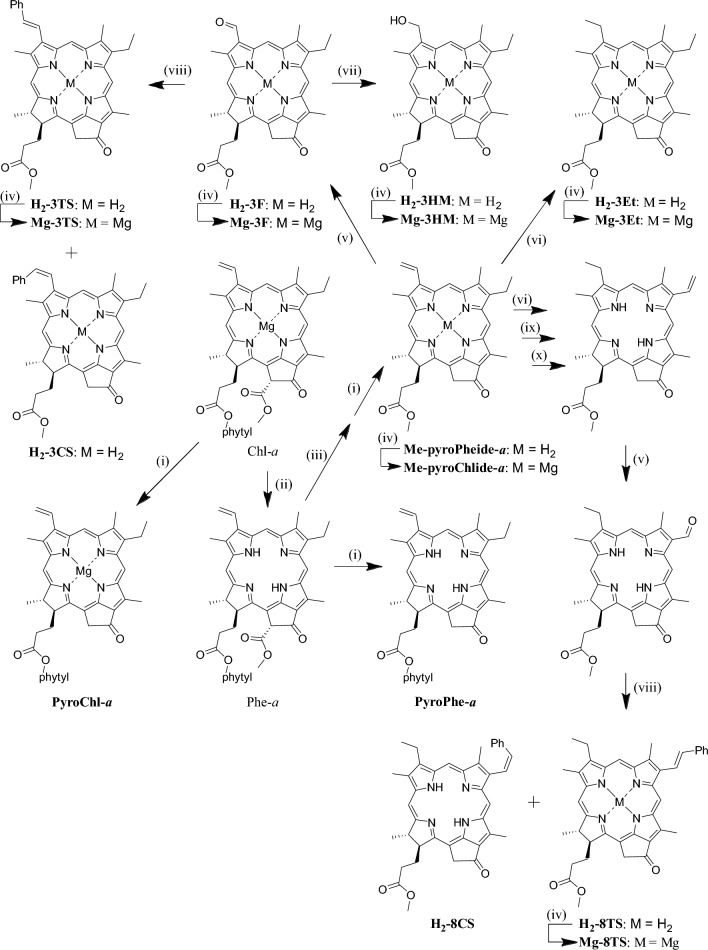
Synthetic routes of magnesium-complexed substrates and authentic demetalated products by chemical modification of Chl-*a*: (i) collidine; (ii) 2% H_2_SO_4_/H_2_O; (iii) 10% H_2_SO_4_/MeOH; (iv) Mg(ClO_4_)_2_, pyridine; (v) NaIO_4_, OsO_4_, AcOH, H_2_O, THF; (vi) H_2_, Pd/C, THF, acetone; (vii) *tert*-BuNH_2_·BH_3_, CH_2_Cl_2_; (viii) PhCH_2_PPh_3_Cl, CH_2_Cl_2_, NaOH, H_2_O; (ix) OsO_4_, C_5_H_5_N, CH_2_Cl_2_, and H_2_S, MeOH; (x) 4-MeC_6_H_4_SO_3_H, C_6_H_6_, CH_2_Cl_2_

Pyrochlorophyll-*a* and metal-free samples were synthesized by chemical modification of Chl-*a* extracted from commercially available *Spirulina* powders. Free bases were metalated by refluxing them in pyridine with magnesium perchlorate (Baum et al. 1964; Ogasawara and Tamiaki 2015) to afford the corresponding magnesium complexes after FCC purification: see Supporting Information for their spectral data. The synthetic routes of chlorophyll derivatives are shown in Fig. 4. Most synthetic procedures were reported in previously reports (Bible et al. 1988; Hirose et al. 2020; Ogasawara et al. 2015; Pandey et al. 1997; Smith et al. 1985; Tamiaki et al. 1996; 1997; 1998; Yagai et al. 1999): see also Supporting Information.

### Activity assay of AbSGR-h in vitro

Chl-*a* derivatives as substrates (20 μM as the final concentration) were dissolved in dimethyl sulfoxide (DMSO, 2.5 μL) and diluted with an aqueous 25 mM Tris–HCl buffer (pH 7.6, 75 μL) and an aqueous 500 mM ethylenediaminetetraacetic acid disodium salt dihydrate (EDTA-2Na) solution (pH 8.0, 2.5 μL). After 5 μM of the AbSGR-h lysate (20 μL) was added to the above substrate solution, the mixture was incubated at 25 °C for 1 h under air in the dark. To stop the enzymatic reaction, 200 μL of acetone was added to the reaction mixture. The resulting protein precipitates were removed by centrifugation (1500 × *g*) for 10 min. Acetone in the supernatant was evaporated under the steam of N_2_. The resulting aqueous solution was subjected to HPLC analysis.

## Results

### Design of Chl-*a* derivative as model substrate for AbSGR-h enzymatic reactions

In vitro AbSGR-h catalyzes the removal of the central Mg in Chl-*a* (Dey et al. 2021). We focused on the C13^2^-methoxycarbonyl group and phytyl ester in the C17-propionate residue of Chl-*a* and synthesized **pyroChl-*a*** that lacks the C13^2^-methoxycarbonyl group and **Me-pyroChlide-*a*** as the methyl esterified form of **pyroChl-*a***. In vitro AbSGR-h enzymatic reactions of the two synthetic substrates were investigated.

**Fig. 5:**
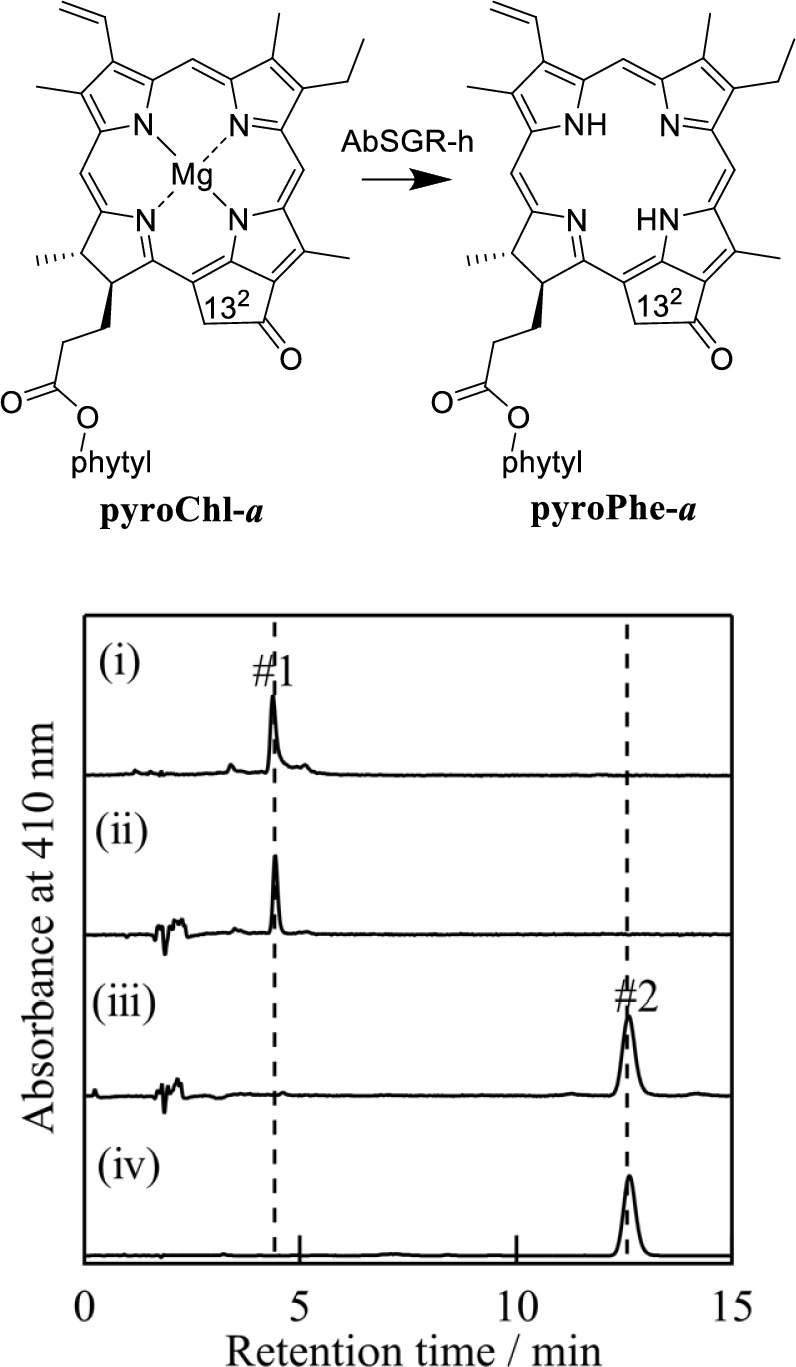
AbSGR-h activity with **pyroChl-*a***. From top to bottom, the HPLC profiles show C13^2^-demethoxycarbonylated **pyroChl-*a*** before (i) and after incubation without (ii) and with AbSGR-h for 1 h (iii) and authentic product **pyroPhe-*a*** (iv): Cosmosil 5C_18_-AR-II, 4.6 ϕ × 150 mm; MeOH/acetone = 65/35 (v/v); 1.0 mL/min. The enzymatic reaction is shown in the activity assay of AbSGR-h in vitro in the Materials and methods section

An AbSGR-h enzymatic reaction similar to that in Chl-***a*** (Dey et al. 2021) was applied to **pyroChl-*a*** (the upper left drawing in Fig. 5). The enzymatic reaction was examined in an aqueous 25 mM Tris–HCl buffer solution containing 2.5% DMSO and 12.5 mM EDTA-2Na at 25 °C in the dark (see Material and methods). After incubation for 1 h, band #1 of the pyroChl-*a* substrate disappeared, and only a single band #2 was detected [comparing (i) and (iii) profiles in Fig. 5 lower]. The on-line visible absorption spectrum of band #2 with Soret/Qx/Qy maxima = 409/609/664 nm differed from that of band #1 (431/618/663 nm), as shown in Figs. S1A/B. The on-line mass spectrum indicated that the product exhibited a parent peak (*m*/*z*) at 813.6, which corresponded to the calculated mass number of protonated pyropheophytin-*a* (**pyroPhe-*a***, Fig. 5 upper, right), i.e., MH^+^,* m*/*z* = 813.5 (MH^+^), as shown in Figs. S1C/D. In addition, the visible absorption and mass spectra and retention time (*t*_R_ = 13.5 min) of band #2 were consistent with those of the authentic product sample of **pyroPhe-*a*** [Fig. 5 lower, (iii)/(iv)]. The results showed that the AbSGR-h enzyme recognized **pyroChl-*a*** (band #1, *t*_R_ = 4.4 min) and demetalated it to **pyroPhe-*a***. Under the aforementioned conditions, **pyroChl-*a*** was fully converted into **pyroPhe-*a***.

**Fig. 6:**
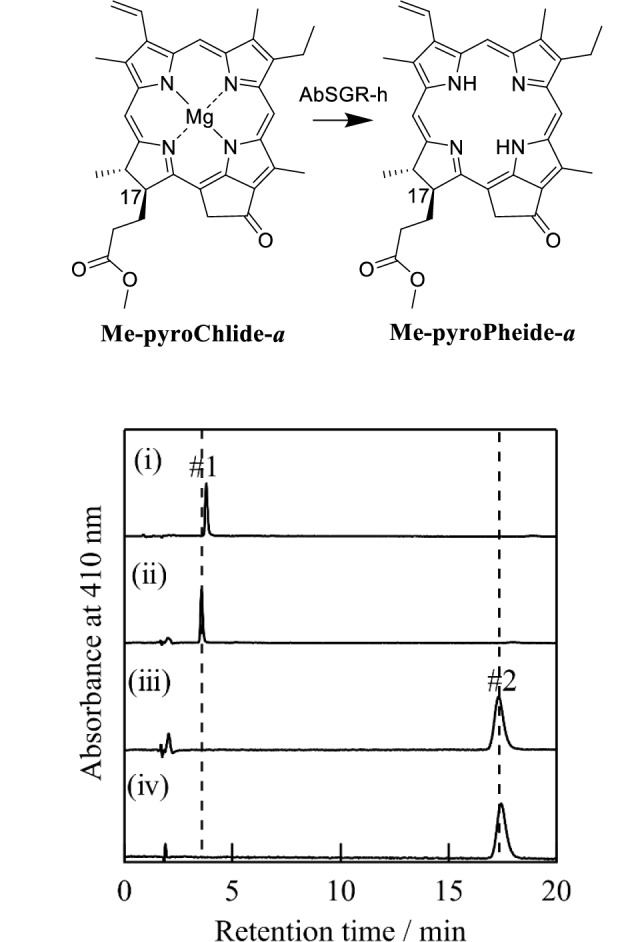
AbSGR-h activity with **Me-pyroChlide-*a***. From top to bottom, the HPLC profiles show C13^2^-demethoxycarbonylated **Me-pyroChlide-*a*** before (i) and after incubation without (ii) and with AbSGR-h for 1 h (iii) and authentic product **Me-pyroPheide-*a*** (iv): Cosmosil 5C_18_-AR-II, 4.6 ϕ × 150 mm; MeOH/H_2_O = 95/5; 1.0 mL/min. The enzymatic reaction is shown in the activity assay of AbSGR-h in vitro in the Materials and methods section

Next, we focused on a phytyl ester in the C17-substituent of Chl-*a*. In our previous report (Obata et al. 2019), the in vitro AbSGR-h enzymatic reaction of Chlide-*a* bearing a free carboxy group in the C17-substituent showed a similar activity as that of Chl-*a* possessing a phytyl ester. Based on the previous study, we examined using methyl pyrochlorophyllide-*a* (**Me-pyroChllide-*a***) as substrate, where the phythyl group at the C17-propionate residue of **pyroChl-*a*** was modified to the methyl ester (see the upper scheme in Fig. 6). Band #1 (*t*_R_ = 3.6 min) of the ***Me-pyroChlide-a*** substrate disappeared, and a new band #2 (*t*_R_ = 18.1 min) was only visible after incubation for 1 h [comparing (i) and (iii) profiles in Fig. 6 lower]. The visible absorption and mass spectra and *t*_R_ value of band #2 were consistent with those of the authentic product methyl pyropheophorbide-*a* (**Me-pyroPheide-*a***, Fig. 6 upper, right) [Fig. 6 lower, (iii)/(iv) and Fig. S2]. The results showed that the AbSGR-h enzymatic reaction completely proceeded for not only the phytyl ester but also the methyl ester.

The above results indicated that the substrate specificities for in vitro AbSGR-h enzymatic reactions were not as high, with tolerance for the removal of the C13^2^-methoxycarbonyl group and the transesterification of phytyl to methyl groups. We found that **Me-pyroChlide-*a*** can serve as a good model substrate for AbSGR-h enzymatic reactions.

### Substrate specificities for AbSGR-h enzymatic reactions of C3-substituted Chl-*a* derivatives

We examined the substrate specificities for the in vitro AbSGR-h enzymatic reactions of synthetic C3-substituted Chl-*a* derivatives, methyl mesopyrochlorophyllide-*a* (**Mg-3Et**), methyl 3-devinyl-3-hydroxymethyl-pyrochlorophyllide-*a* (**Mg-3HM**), and methyl pyrochlorophyllide-*d* (**Mg-3F**) with the C3-ethyl, hydroxymethyl, and formyl groups, respectively, on the basis of the model substrate **Me-pyroChlide-*a***. The AbSGR-h enzymatic reaction of the C3-formyl substitute **Mg-3F** (Fig. S3 upper, left) was investigated under the aforementioned conditions. After incubation for 1 h, band #1 (*t*_R_ = 2.6 min) of the **Mg-3F** substrate decreased, and a new band #2 (*t*_R_ = 15.2 min) appeared [comparing (i) and (iii) profiles in Fig. S3]. Band #2 was assigned to methyl pyropheophorbide-*d* (**H**_**2**_**-3F**), which lacks the central Mg, by comparing the on-line visible and mass spectra and *t*_R_ of the authentic product sample [Fig. S3 (iii)/(iv) and Fig. S4]. The partial transformation of **Mg-3F** to **H**_**2**_**-3F** showed that the AbSGR-h enzymatic reaction was less effective for the substrate with an electron-withdrawing formyl group at the C3-position than for the other substrates with the C3-vinyl group.

Under the same conditions mentioned above, the AbSGR-h-catalyzed reaction of the C3-ethylated substitute **Mg-3Et** was investigated (the upper scheme in Fig. S5). After incubation for 1 h, band #1 (*t*_R_ = 3.9 min) of the **Mg-3Et** substrate disappeared, and sole methyl mesopyropheophorbide-*a* (**H**_**2**_**-3Et**) was detected as the product [*t*_R_ = 15.1 min, Fig. S5 lower, (iii)/(iv) and Fig. S6]. These results indicated that the AbSGR-h catalyzed the demetalation of **Mg-3Et** similarly as in **Mg-pyroChlide-*a***, whereas the C3-ethyl group was more conformationally flexible than the C3-vinyl group.

The AbSGR-h enzymatic reaction of the C3-hydroxymethylated substitute **Mg-3HM** was examined under the aforementioned conditions (see the upper scheme in Fig. S7). After incubation for 1 h, band #1 (*t*_R_ = 5.0 min) of the **Mg-3HM** substrate disappeared, and new band #2 of methyl 3-devinyl-3-hydroxymethyl-pyropheophorbide-*a* (**H**_**2**_**-3HM**) was detected as a demetalation product (*t*_R_ = 16.1 min, Fig. S7 lower, (iii)/(iv) and Fig. S8). These results showed that the AbSGR-h enzymatic reaction was not significantly affected by the hydrophilic group in the C3-substituent.

C3-substituted substrates with formyl, ethyl, and hydroxymethyl groups were recognized and demetalized by AbSGR-h. The results indicated that the enzyme could recognize such C3-functional groups and that the catalytic reactivity was partially dependent on their electron-withdrawing factors.

### Proposed position of Chl-*a* inside the active site of AbSGR-h

The crystal structure of AbSGR-h has been reported (Dey et al. 2022). However, the precise supramolecular structure of a complex of the enzyme with a substrate has not yet been reported. Therefore, it is unclear how Chl-*a* is positioned inside the active site of SGR. To gain more clarity, we examined the AbSGR-h enzymatic activity of synthetic Chl-*a* derivatives with the C3- or C8-styryl group as a bulky functional group.

The AbSGR-h enzymatic reaction of the C3-styrylated substitute, methyl *trans*-3^2^-phenyl-chlorophyllide-*a* (**Mg-3TS**), was examined under the aforementioned conditions (see the upper scheme in Fig. S9). Band #1 of the **Mg-3TS** (*t*_R_ = 3.2 min) substrate decreased, and a new band #2 (*t*_R_ = 16.8 min) was detected after incubation for 1 h [(i)–(iii) profiles in Fig. S9]. The on-line visible/mass spectra and *t*_R_ of band #2 (*t*_R_ = 16.7 min) were consistent with those of the free base **H**_**2**_**-3TS** as the authentic product sample [Fig. S9 lower, (iii)/(iv) and Fig. S10]. These results indicated that AbSGR-h recognized **Mg-3TS** and catalyzed its demetalation. Furthermore, the reactivity of **Mg-3TS** was lower than that of **Me-pyroChlide-*a***.

Next, the AbSGR-h enzymatic reaction of the C8-styrylated counterpart, methyl 8-deethyl-8-*trans*-styryl-mesopyrochlorophyllide-*a* (**Mg-8TS**), was investigated under the aforementioned conditions (the upper scheme in Fig. S11). After incubation for 1 h, band #1 (*t*_R_ = 3.2 min) of the **Mg-8TS** substrate decreased only slightly, and a new band #2 (*t*_R_ = 18.1 min) of **H**_**2**_**-8TS** was slightly observed as a demetalation product [the (iii) profile in Fig. S11 and S12].

The above results showed that the AbSGR-h enzyme was tolerant to the Chl-*a* derivative styrylated at the C3-position on the A-ring and that enzymatic demetalation partially proceeded. In contrast, the regioisomeric Chl-*a* derivative styrylated at the C8-position on the B-ring is difficult to recognize by its enzyme. These results indicate that demetalation catalyzed by AbSGR-h depends on the configuration and position of the substrate inside its active site. Therefore, the position of the B-ring in Chl-*a* inside the AbSGR-h active site is important for enzymatic demetalation.

## Discussion

**Fig. 7:**
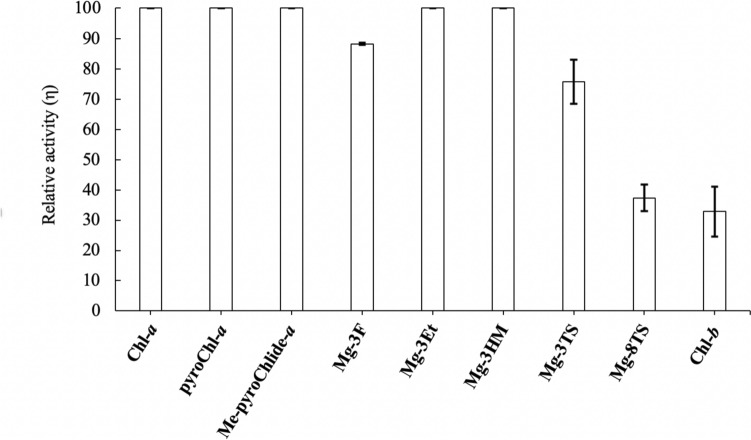
Relative activity of Chl derivatives by AbSGR-h. This is illustrated by the relative reactivity of each Chl derivative compared with Chl-*a*, which is assumed to be 100. AbSGR-h activity with Chl-*b* is shown in Fig. S13. The enzymatic reaction activity was determined from the band areas in the RP-HPL chromatograms: {1 − [substrate after SGR reaction]/[substrate before SGR reaction]} × 100. Data are average values from three independent experiments. Bars represent mean ± standard deviation

The reactivity of Chl-*a* derivatives with various C3-substituents for in vitro AbSGR-h dechelations showed that the substrate **Mg-3F** with the electron-withdrawing formyl group exhibited lower reactivity (Fig. 7): relative activity η = 70% (**Mg-3F**) < 100% (**Mg-3V**/**3Et**/**3HM**). In particular, the demetalation of **Mg-3V** under acidic conditions was rapider than that of **Mg-3F** (Saga et al. 2012; Saga and Tamiaki 2012; Mazaki et al. 1992). The acidic demetalation order was consistent with that of AbSGR-h enzymatic demetalation. The electron-withdrawing group reduced the electron density on the inner nitrogen atom of the cyclic tetrapyrrole, retarding the protonation required for dechelation. A similar electronic effect was observed for both dechelatase-catalyzed and acid-promoted reactions.

In addition to the electronic factor, a steric effect was observed for the AbSGR-h reaction. Enzymatic demetalation of the Chl-*a* derivative **Mg-3TS** with the bulky C3-styryl group on the A-ring partially proceeded. The AbSGR-h enzyme significantly less catalyzed the demetalation of its isomeric **Mg-8TS** with the C8-styryl group on the B-ring: η = 40% (**Mg-8TS**) < 80% (**Mg-3TS**). These results are supported by a previously reported docking simulation of Chl-*a* with AbSGR-h in which the B-ring in Chl-*a* is positioned deeper in its active site than the A-ring (Dey et al. 2022).

The substrate specificity of enzymes is the fundamental basis for developing metabolic pathways. In each step of a metabolic pathway, enzymes catalyze the reaction of the accurate substrate. If enzymes accept the wrong molecule as the substrate, the metabolic pathway is disturbed because the essential molecule is not produced, and a useless product can affect other metabolic pathways. In the Chl degradation pathway, Pheide-*a* oxygenase (PAO) substrate specificity is critical to accurately degrade Chl. PAO oxidatively opens only the Chl-*a*-derived tetrapyrrole ring (Hörtensteiner et al. 1995) but cannot oxidatively cleave the C7-formyl derivative Chl-*b*. This is why Chl-*b* reverses to Chl-*a* and then degrades in the same pathway as Chl-*a*. Chl-*b*-overproducing transformant plants accumulate Pheide-*b*, possibly because PAO cannot oxygenate it and the central Mg of Chl-*b* is nonenzymatically removed. These transformant plants exhibit necrotic damage (Shimoda et al. 2012). This damage may be caused by the accumulation of phototoxic Pheide-*b* derivatives. These observations indicate that the SGR of green plants should not remove the central Mg from Chl-*b*. During the evolutionary process, SGR substrate specificity increases, and Chl-*b* becomes the unfavorable substrate. The relative reactivity of Chl-*b* at η = 30% suggests that, despite substrates with electron-withdrawing groups being less favorable for AbSGR-h (Fig. 7), AbSGR-h may still be able to extract Mg from such molecules. In vitro, plant SGR cannot extract Mg from Chl-*b* (Obata et al. 2019). This is likely influenced by the abundance of hydrophobic amino acids near the plant SGR active site. The docking simulations of AbSGR-h with Chl-*a* revealed that the interior was filled with hydrophobic amino acids compared with the surface of AbSGR-h (Fig. S14A). By contrast, an interior of plant SGR has more hydrophobic environment (Fig. S14B), which would be contributed by different hydrophobic amino acid residues (Dey et al. 2021). Based on this study that the Chls bind with the B-ring facing AbSGR-h, the hydrophobic difference between plant SGR and AbSGR-h could be generated during the evolutionary process. This evolution from AbSGR-h to plant SGR may selectively prevent the reaction of Chl-*b*, where the hydrophilic formyl group is bound to the B-ring, thereby preventing the accumulation of difficult-to-degrade Pheide-*b*.

## Conclusion

We systematically prepared various Mg-chelated substrates for SGR that lack the methoxycarbonyl group at the C13^2^-position. The synthetic substrates are more chemically stable and can be handled more easily than natural Chl substrates, due to the absence of the enolizable and allomerizable keto-ester groups. The substitution effects at the C3- and C8-positions of the substrates on in vitro AbSGR-h dechelation were revealed, i.e., the electronic and steric factors regulated the reactivities of the substrates. The electron-withdrawing and sterically demanding groups at the peripheral positions suppressed the removal of the central Mg of the substrate core. The steric effect was more effective for the C8-position than for the C3-position, suggesting that SGR preferentially bonded to the B-ring of Chl.

### Supplementary Information

Below is the link to the electronic supplementary material.

Supplementary file1 (PDF 1666 KB)

## Data Availability

Data will be made available on request.

## Abbreviations

3Et: C3-ethyl-Chl derivative
3F: C3-formyl-Chl derivative
3HM: C3-hydroxymethyl-Chl derivative
3TS: C3-*trans*-styryl-Chl derivative
8TS: C8-*trans*-styryl-Chl derivative
AbSGR-h: *Anaerolineae* bacterium SM23_63 Stay-Green homolog
Chl: Chlorophyll
Chlide: Chlorophyllide
DMSO: Dimethyl sulfoxide
EDTA-2Na: Ethylenediaminetetraacetic acid disodium salt dihydrate
FCC: Flash column chromatography
HPLC: High-performance liquid chromatography
LC–MS: Liquid chromatography–mass spectrometry
Me: Methyl
PAO: Pheophorbide-*a* oxygenase
Phe: Pheophytin
Pheide: Pheophorbide

## References

[CR1] Baum SJ, Burnham BF, Robert A, Plane RA (1964). Studies on the biosynthesis of chlorophyll: chemical incorporation of magnesium into porphyrins. Proc Natl Acad Sci USA.

[CR2] Bible KC, Buytendorp M, Zierath PD, Rinehart KL (1988). Tunichlorin: a nickel chlorin isolated from the Caribbean tunicate *Trididemnum solidum*. Proc Natl Acad Sci USA.

[CR3] Dey D, Dhar D, Fortunato H, Daichi O, Tanaka A, Tanaka R, Basu S, Ito H (2021). Insights into the structure and function of the rate-limiting enzyme of chlorophyll degradation through analysis of a bacterial Mg-dechelatase homolog. Comp Struct Biotech J.

[CR4] Dey D, Nishijima M, Tanaka R, Kurisu G, Tanaka H, Ito H (2022). Crystal structure and reaction mechanism of a bacterial Mg-dechelatase homolog from the Chloroflexi *Anaerolineae*. Protein Sci.

[CR5] Hirose M, Teramura M, Harada J, Tamiaki H (2020). BciC-catalyzed C13^2^-demethoxycarbonylation of metal pheophorbide *a* alkyl esters. ChemBioChem.

[CR6] Hirose M, Tsukatani Y, Harada J, Tamiaki H (2022). Characterization of regioisomeric diterpenoid tails in bacteriochlorophylls produced by geranylgeranyl reductase from *Halorhodospira halochloris* and *Blastochloris viridis*. Photosynth Res.

[CR7] Hörtensteiner S, Vicentini F, Matile P (1995). Chlorophyll breakdown in senescent cotyledons of rape, *Brassica napus* L.: enzymatic cleavage of phaeophorbide a in vitro. New Phytol.

[CR8] Kräutler B (2019). Chlorophyll breakdown – How chemistry has helped to decipher a striking biological Eenigma. Synlett.

[CR10] Mazaki H, Watanabe T, Takahashi T, Struck A, Scheer H (1992). Pheophytinization of eight chlorophyll derivatives in aqueous acetone. Bull Chem Soc Jpn.

[CR11] Obata D, Takabayashi A, Tanaka R, Ito H (2019). Horizontal transfer of promiscuous activity from nonphotosynthetic bacteria contributed to evolution of chlorophyll degradation pathway. Mol Biol Evol.

[CR12] Ogasawara S, Tamiaki H (2015). Synthesis of methyl (13^2^*R*/*S*)-alkyl-pyropheophorbide *a* and a non-epimerized chlorophyll *a* mimic. Bioorg Med Chem.

[CR13] Pandey RK, Isaac M, Ian MacDonald, Medforth CJ, Senge MO, Doughterty TJ, Smith KM (1997). Pinacol–pinacolone rearrangements in *vic*-didehydroxychlorins and bacteriochlorins: effect of substituents at the peripheral positions. J Org Chem.

[CR14] Saga Y, Tamiaki H (2012). Demetalation of chlorophyll pigments. Chem Biodivers.

[CR15] Saga Y, Kobashiri Y, Sadaoka K (2012). Systematic analysis of the demetalation kinetics of zinc chlorophyll derivatives possessing different substituents at the 3-position: effects of the electron-withdrawing and electron-donating strength of peripheral substituents. Inorg Chem.

[CR16] Shimoda Y, Ito H, Tanaka A (2012). Conversion of chlorophyll *b* to chlorophyll *a* precedes magnesium dechelation for protection against necrosis in Arabidopsis. Plant J.

[CR17] Shimoda Y, Ito H, Tanaka A (2016). Arabidopsis *STAY-GREEN*, Mendel’s Green cotyledon gene, encodes magnesium-dechelatase. Plant Cell.

[CR18] Smith KM, Goff DA, Simpson DJ (1985). *Meso* substitution of chlorophyll derivatives: direct route for transformation of bacteriopheophorbides *d* into bacteriopheophorbides *c*. J Am Chem Soc.

[CR19] Tamiaki H, Kouraba M (1997). Synthesis of chlorophyll-*a* homologs by Wittig and Knoevenagel reactions with methyl pyropheophorbide-*d*. Tetrahedron.

[CR20] Tamiaki H, Amakawa M, Shimono Y, Tanikaga R, Holzwarth AR, Schaffner K (1996). Synthetic zinc and magnesium chlorin aggregates as models for supramolecular antenna complexes in chlorosomes of green photosynthetic bacteria. Photochem Photobiol.

[CR21] Tamiaki H, Takeuchi S, Tsudzuki S, Miyatake T, Tanikaga R (1998). Aggregation of synthetic zinc chlorins with several esterified alkyl chains as models of bacteriochlorophylls-*c* homologs. Tetrahedron.

[CR22] Yagai S, Tomohiro M, Tamiaki H (1999). Self-assembly of synthetic 8^1^-hydroxy-chlorophyll analogues. Tetrahedron.

