# Supplementary material for: In vitro demetalation of central magnesium in various chlorophyll derivatives using Mg-dechelatase homolog from the chloroflexi Anaerolineae
Source: Photosynth Res. 2024 Mar 26;160(1):45–53. doi: 10.1007/s11120-024-01088-4 (PMC11006732; doi:10.1007/s11120-024-01088-4)
Supplement: Supplementary file 1 — Supplementary file1 (PDF 1666 KB) [file 11120_2024_1088_MOESM1_ESM.pdf]

Supporting Information

In vitro demetallation of central magnesium in various chlorophyll derivatives by Mg-dechelatase homolog from the Chloroflexi *Anaerolineae*

Soma Sato[1,2,3] • Mitsuaki Hirose[1,4] • Ryouichi Tanaka[3] • Hisashi Ito[3] • Hitoshi Tamiaki[1,*]

1 Graduate School of Life Sciences, Ritsumeikan University, Kusatsu, Shiga 525-8577, Japan

2 Graduate School of Environment Science, Hokkaido University, N10 W5, Sapporo 060-0810, Japan

3 Institute of Low Temperature Science, Hokkaido University, N19 W8, Sapporo 060-0819, Japan

4 Department of Science and Technology, Seikei University, Tokyo, 180-8633, Japan

* Corresponding author, e-mail: tamiaki@fc.ritsumei.ac.jp

Contents

Methods

Spectral data of Chl-*a* derivatives (magnesium complexes) S3

Synthesis of Chl-*a* derivatives (free bases) S4–S5

Figures

On-line absorption and mass spectra of **pyroChl-*a*** and **pyroPhe-*a*** (Fig. S1) S6

On-line absorption and mass spectra of **Me-pyroChlide-*a*** and **Me-pyroPheide-*a*** (Fig. S2) S7

HPLC elution profiles for AbSGR-h activity with **Mg-3F** (Fig. S3) S8

On-line absorption and mass spectra of **Mg-3F** and **H_2-3F** (Fig. S4) S9

HPLC elution profiles for AbSGR-h activity with **Mg-3Et** (Fig. S5) S10

On-line absorption and mass spectra of **Mg-3Et** and **H_2-3Et** (Fig. S6) S11

HPLC elution profiles for AbSGR-h activity with **Mg-3HM** (Fig. S7) S12

On-line absorption and mass spectra of **Mg-3HM** and **H_2-3HM** (Fig. S8) S13

HPLC elution profiles for AbSGR-h activity with **Mg-3TS** (Fig. S9) S14

On-line absorption and mass spectra of **Mg-3TS** and **H_2-3TS** (Fig. S10) S15

HPLC elution profiles for AbSGR-h activity with **Mg-8TS** (Fig. S11) S16

On-line absorption and mass spectra of **Mg-8TS** and **H_2-8TS** (Fig. S12) S17

HPLC elution profiles for AbSGR-h activity with Chl-*b* (Fig. S13) S18

Docking simulation of AbSGR-h with Chl-*a* (Fig. S14) S19

References S20–S21

Spectral data of Chl-*a* derivatives (magnesium complexes)

Pyrochlorophyll-a ***(pyroChl-a)***: Vis (MeOH/acetone = 65/35) λ_{max}/nm = 431 (relative intensity 1.00), 618 (0.20), 663 (0.90); MS (APCI) found: *m/z* 835.6, calcd for $C_{53}H_{71}N_4O_3Mg^+$: MH^+, 835.5.

Methyl pyrochlorophyllide-a ***(Me-pyroChlide-a)***: Vis (MeOH/H_2O = 95/5) λ_{max}/nm = 431 (relative intensity 1.00), 617 (0.23), 665 (0.99); MS (APCI) found: *m/z* 571.2, calcd for $C_{34}H_{35}N_4O_3Mg^+$: MH^+, 571.3.

Methyl pyrochlorophyllide-d ***(Mg-3F)***: Vis (MeOH/H_2O = 97/3) λ_{max}/nm = 401 (relative intensity 0.71), 454 (0.75), 698 (1.00); MS (APCI) found: *m/z* 573.2, calcd for $C_{33}H_{33}N_4O_4Mg^+$: MH^+, 573.2.

Methyl mesopyrochlorophyllide-a ***(Mg-3Et)***: Vis (MeOH/H_2O = 95/5) λ_{max}/nm = 426 (relative intensity 1.00), 607 (0.23), 653 (0.97); MS (APCI) found: *m/z* 573.3, calcd for $C_{34}H_{37}N_4O_3Mg^+$: MH^+, 573.3.

Methyl 3-devinyl-3-hydroxymethyl-pyrochlorophyllide-a ***(Mg-3HM)***: Vis (MeOH/H_2O = 85/15) λ_{max}/nm = 429 (relative intensity 0.96), 614 (0.23), 658 (1.00); MS (APCI) found: *m/z* 575.3, calcd for $C_{33}H_{35}N_4O_4Mg^+$: MH^+, 575.3.

Methyl trans-3^2-phenyl-pyrochlorophyllide-a ***(Mg-3TS)***: Vis (MeOH/acetone = 99.8/0.2) λ_{max}/nm = 436 (relative intensity 1.00), 624 (0.24), 672 (0.95); MS (APCI) found: *m/z* 647.3, calcd for $C_{40}H_{39}N_4O_3Mg^+$: MH^+, 647.3.

Methyl 8-deethyl-8-trans-styryl-mesopyrochlorophyllide-a ***(Mg-8TS)***: Vis (MeOH/acetone = 99.8/0.2) λ_{max}/nm = 446 (relative intensity 1.00), 612 (0.16), 654 (0.69); MS (APCI) found: *m/z* 647.3, calcd for $C_{40}H_{39}N_4O_3Mg^+$: MH^+, 647.3.

Synthesis of Chl-*a* derivatives (free bases)

*Synthesis of pyropheophytin-a (**pyroPhe-a**)*: see Hirose et al. (2020) and Kashiyama et al. (2012).

*Synthesis of methyl pyropheophorbide-a (**Me-pyroPheide-a**)*: see Smith et al. (1985).

*Synthesis of methyl pyropheophorbide-d (**H_2-3F**)*: see Bible et al. (1988), Pandey et al. (1997), and Tamiaki et al. (1996).

*Synthesis of methyl mesopyropheophorbide-a (**H_2-3Et**)*: see Smith et al. (1985) and Tamiaki et al. (2013).

*Synthesis of methyl 3-devinyl-3-hydroxymethyl-pyropheophorbide-a (**H_2-3HM**)*: see Tamiaki et al. (1996).

*Synthesis of methyl trans-3^2-phenyl-pyropheophorbide-a (**H_2-3TS**)*: see Tamiaki and Kouraba (1997).

*Synthesis of methyl 8-deethyl-8-trans-styryl-mesopyropheophorbide-a (**H_2-8TS**)*

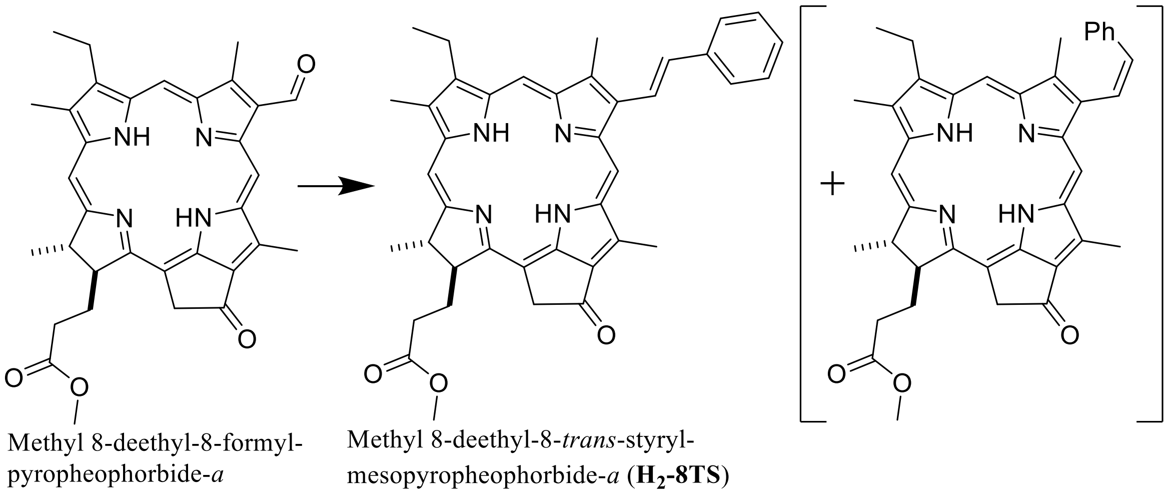

Methyl 8-deethyl-8-formyl-pyropheophorbide-*a*

Methyl 8-deethyl-8-*trans*-styryl-mesopyropheophorbide-*a* (**H_2-8TS**)

Based on the synthesis of **H_2-3TS** with minor modifications (Tamiaki and Kouraba 1997), methyl 8-deethyl-8-formyl-pyropheophorbide-*a* (31.8 mg, 57.7 μmol) (Tamiaki et

al. 2008, 2016) in CH_2Cl_2 (7 mL) was reacted with benzyltriphenylphosphonium chloride (24.6 mg, 63.3 μmol) in an aqueous NaOH solution (7 mg/3 mL) afforded a 5:4 mixture of **H_2-8TS** and its *cis*-isomer (17.8 mg, 28.5 μmol, 49%) as a brown solid. A portion of the regioisomeric mixture was separated by HPLC (Cosmosil $5C_{18}$-AR-II, 10 ϕ × 250 mm, MeOH/acetone = 90/10, 5.0 mL/min) to give a **H_2-8TS**: mp 75–80 °C; ^{1}H NMR ($CDCl_3$, 600 MHz) δ/ppm = 9.71 (1H, s, 10-H), 9.30 (1H, s, 5-H), 8.49 (1H, s, 20-H), 8.35 (1H, d, J = 17 Hz, 8^1-H), 7.53 (1H, d, J = 17 Hz, 8^2-H), 7.87 (2H, d, J = 7 Hz, 2-, 6-H of 8^2-Ph), 7.55 (2H, t, J = 7 Hz, 3-, 5-H of 8^2-Ph), 7.42 (1H, t, J = 7 Hz, 4-H of 8^2-Ph), 5.26, 5.10 (each 1H, d, J = 20 Hz, 13^1-CH_2), 4.48 (1H, dq, J = 8, 2, Hz, 18-H), 4.29 (1H, dt, J = 9, 3 Hz, 17-H), 3.86 (2H, q, J = 8 Hz, 3-CH_2), 3.66 (3H, s, 12-CH_3), 3.61 (3H, s, 17-$COOCH_3$), 3.46 (3H, s, 2-CH_3), 3.30 (3H, s, 7-CH_3), 2.74–2.68, 2.61–2.53, 2.36–2.24 (1H+1H+2H, m, 17-CH_2CH_2), 1.81 (3H, d, J = 7 Hz, 18-CH_3), 1.75 (3H, t, J = 8 Hz, 3^1-CH_3), 0.52, –1.64 (each 1H, br-s, NH × 2); Vis (CH_2Cl_2) λ_{max}/nm = 422 (relative intensity, 1.00), 512 (0.07), 548 (0.02), 603 (0.06), 660 (0.35); HRMS (APCI) found: *m/z* 625.3158, calcd for $C_{40}H_{41}N_4O_3^+$: MH^+, 625.3173.

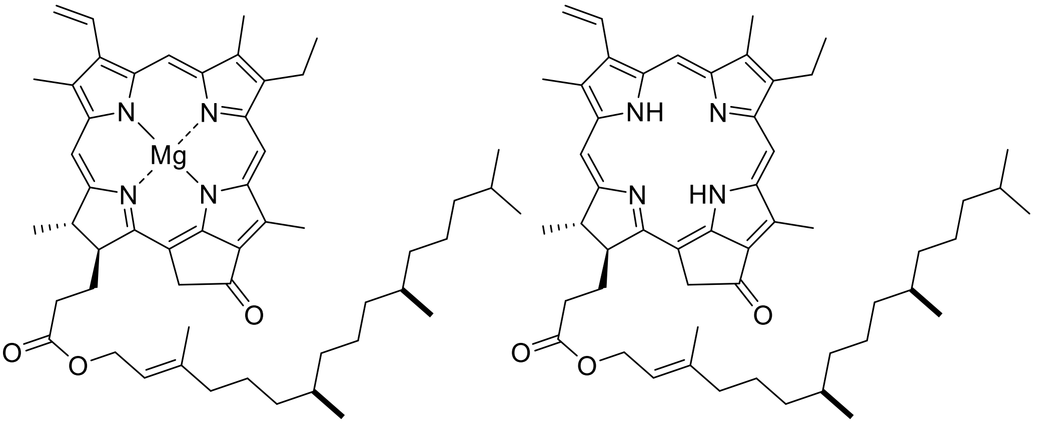

pyroChl-*a*

Calcd for $C_{53}H_{71}N_4O_3Mg^+$; 835.5371

pyroPhe-*a*

Calcd for $C_{53}H_{73}N_4O_3^+$; 813.5677

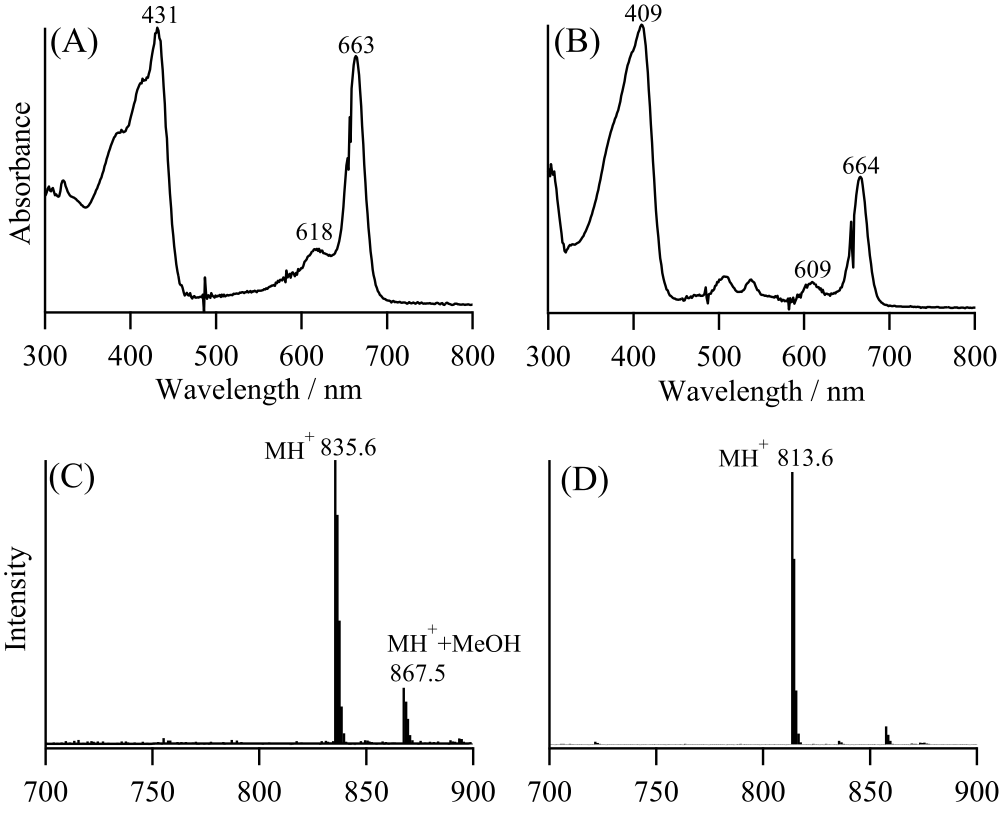

Fig. S1 Chemical structures (upper) of **pyroChl-*a*** (left) and **pyroPhe-*a*** (right). On-line UV-vis spectra (middle) of **pyroChl-*a*** (A) and **pyroPhe-*a*** (B). On-line MS spectra (lower) of **pyroChl-*a*** (C) and **pyroPhe-*a*** (D)

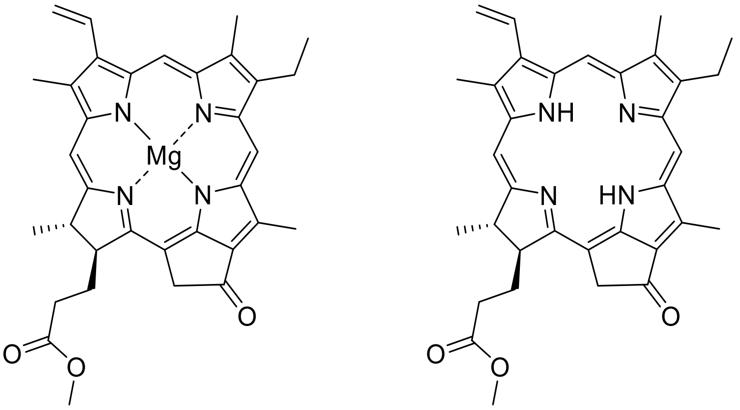

Me-pyroChlide-*a* | **Me-pyroPheide-*a***

Calcd for $C_{34}H_{35}N_4O_3Mg^+$; 571.2554 | Calcd for $C_{34}H_{37}N_4O_3^+$; 549.2860

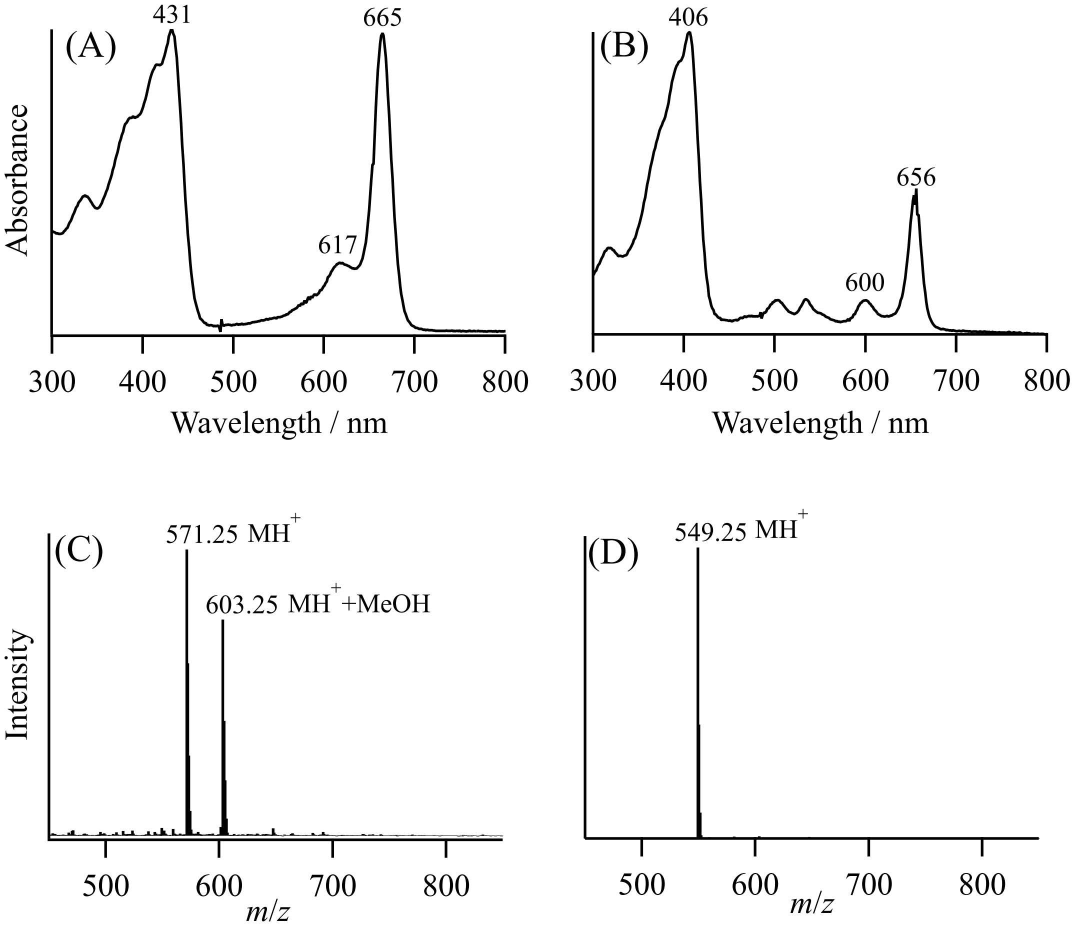

Fig. S2 Chemical structures (upper) of **Me-pyroChlide-*a*** (left) and **Me-pyroPheide-*a*** (right). On-line UV-vis spectra (middle) of **Me-pyroChlide-*a*** (A) and **Me-pyroPheide-*a*** (B). On-line MS spectra (lower) of **Me-pyroChlide-*a*** (C) and **Me-pyroPheide-*a*** (D)

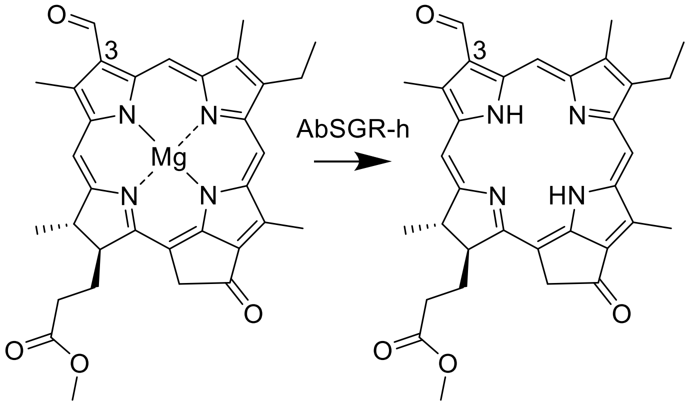

Mg-3F **H_2-3F**

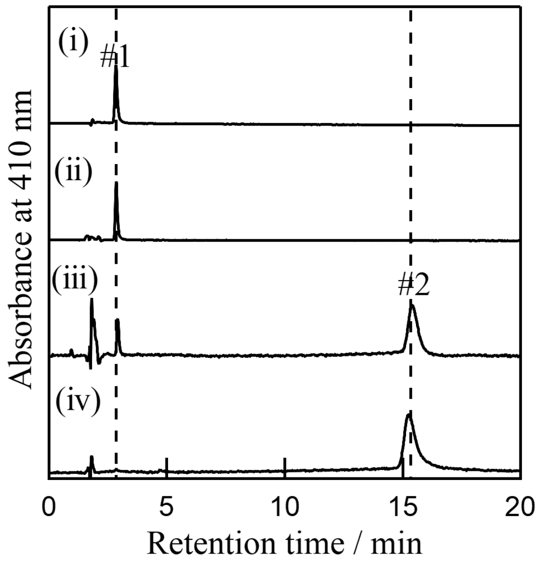

Fig. S3 AbSGR-h activity with **Mg-3F**. From top to bottom, the HPLC profiles show C3-formylated **Mg-3F** before (i) and after incubation without (ii) and with AbSGR-h for 1 h (iii) and authentic product **H_2-3F** (iv): Cosmosil 5C_{18}-AR-II, 4.6 ϕ × 150 mm; MeOH/H_2O = 97/3 (v/v); 1.0 mL/min. The enzymatic reaction is shown in the activity assay of AbSGR-h *in vitro* in the Materials and methods section.

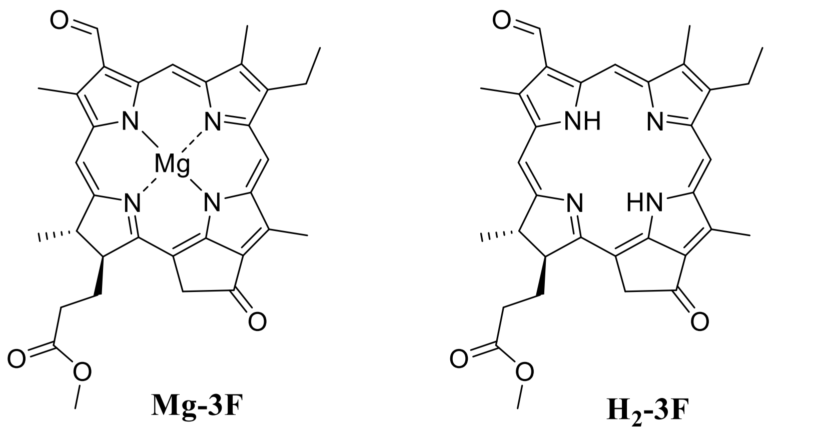

Mg-3F **H_2-3F**

Calcd for $C_{33}H_{33}N_4O_4Mg^+$; 573.2347 Calcd for $C_{33}H_{35}MgN_4O_4^+$; 551.2653

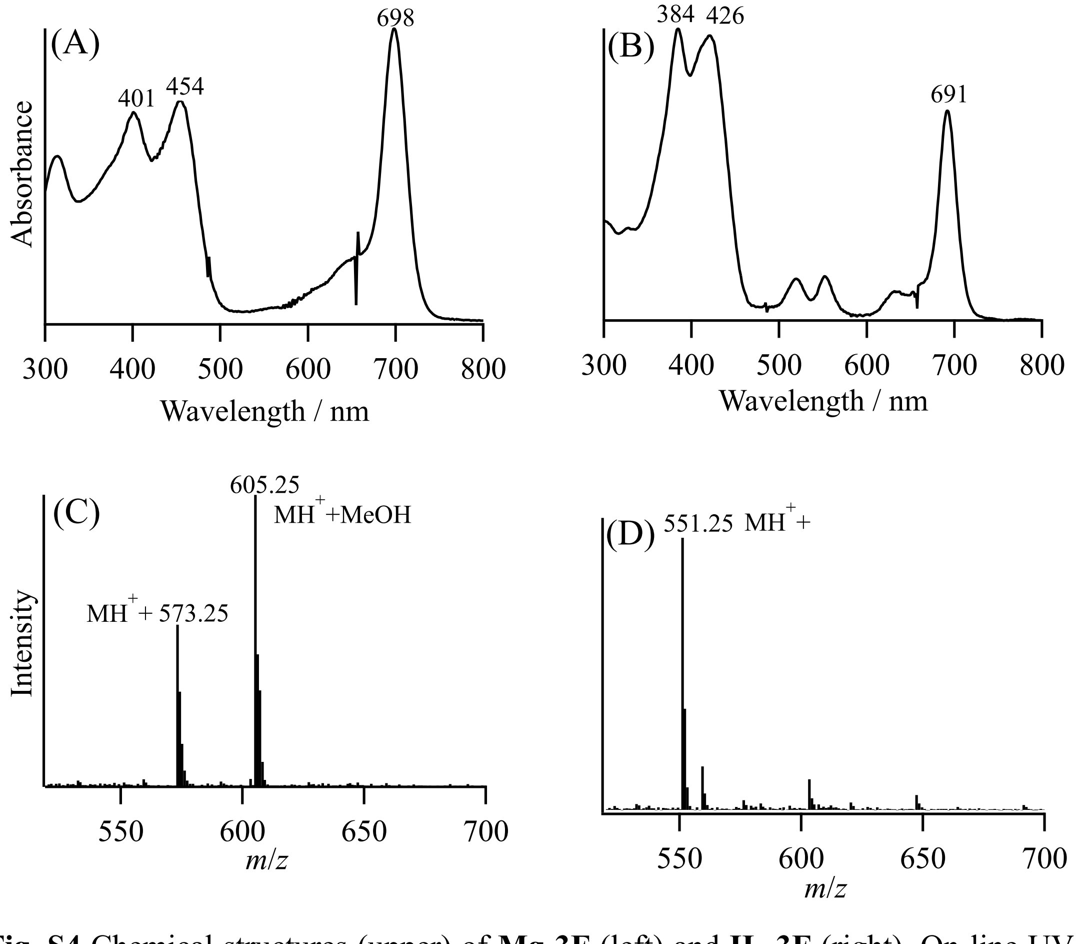

Fig. S4 Chemical structures (upper) of **Mg-3F** (left) and **H_2-3F** (right). On-line UV-vis spectra (middle) of **Mg-3F** (A) and **H_2-3F** (B). On-line MS spectra (lower) of **Mg-3F** (C) and **H_2-3F** (D)

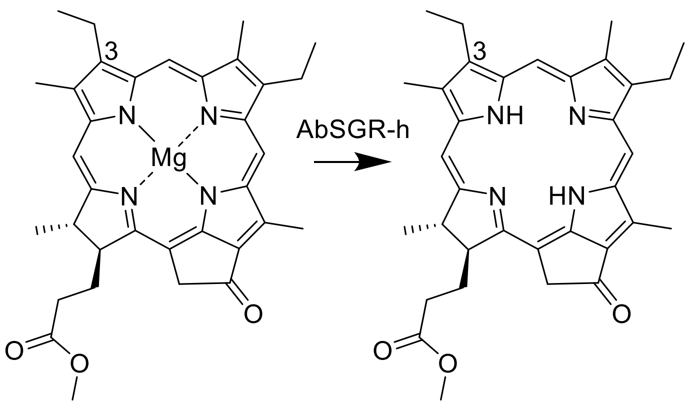

Mg-3Et **H_2-3Et**

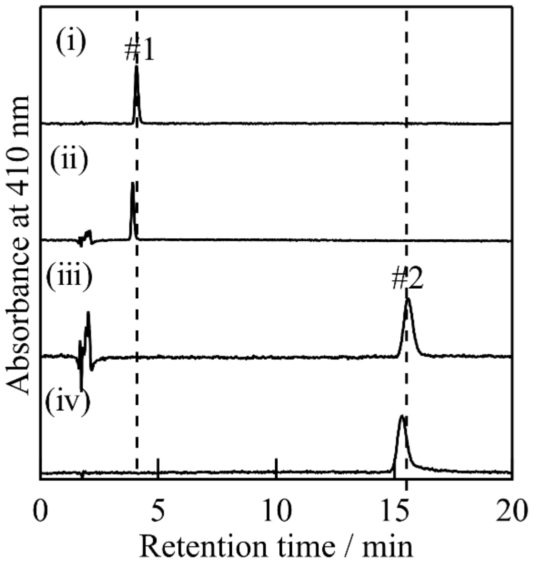

Fig. S5 AbSGR-h activity with **Mg-3Et**. From top to bottom, the HPLC profiles show C3-ethylated **Mg-3Et** before (i) and after incubation without (ii) and with AbSGR-h for 1 h (iii) and authentic product **H_2-3Et** (iv): Cosmosil $5C_{18}$-AR-II, 4.6 ϕ × 150 mm); MeOH/H_2O= 95/5 (v/v); 1.0 mL/min. The enzymatic reaction is shown in the activity assay of AbSGR-h *in vitro* in the Materials and methods section.

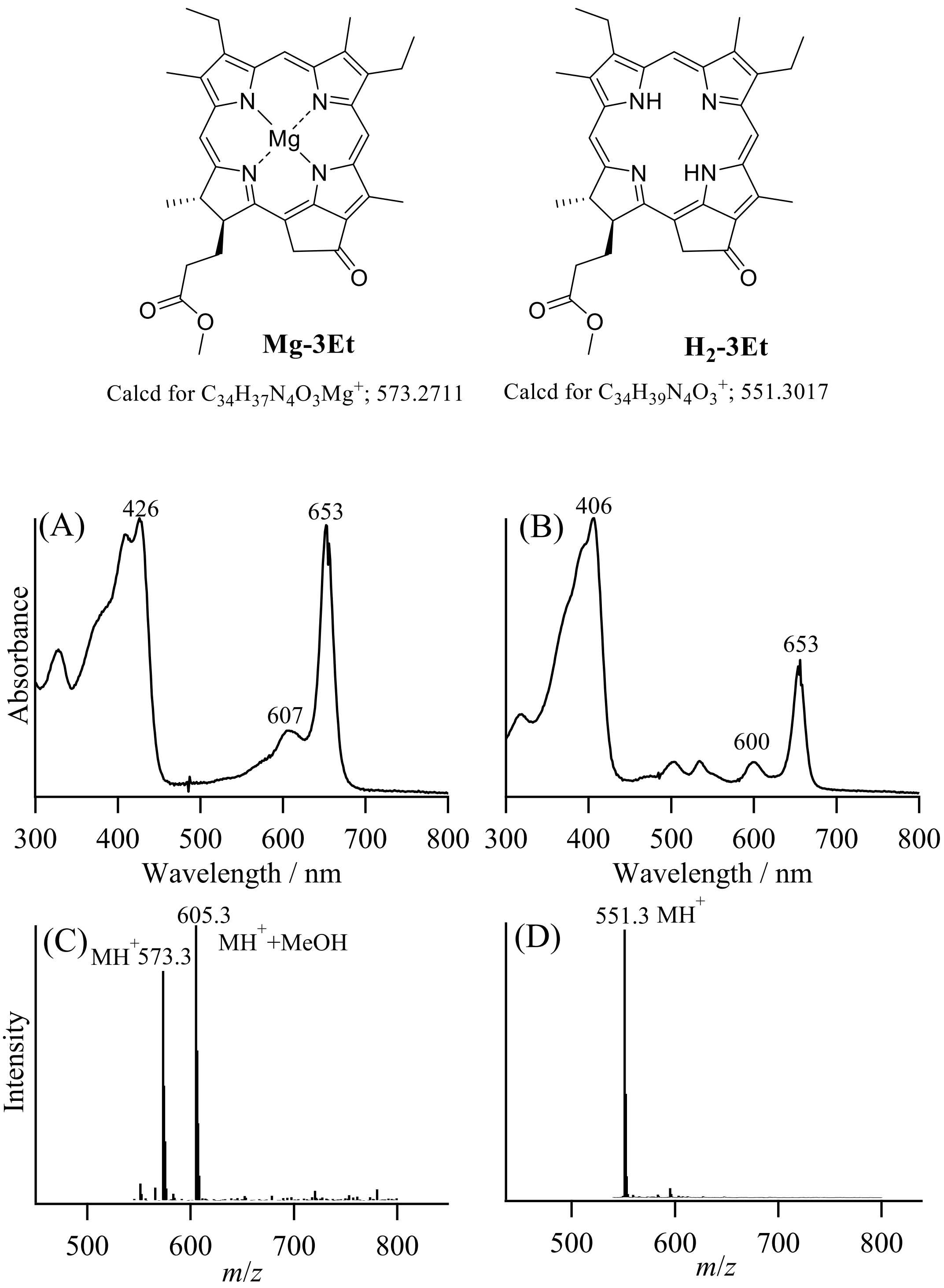

Calcd for $C_{34}H_{37}N_4O_3Mg^+$; 573.2711 Calcd for $C_{34}H_{39}N_4O_3^+$; 551.3017

Fig. S6 Chemical structures (upper) of **Mg-3Et** (left) and **H_2-3Et** (right). On-line UV-vis spectra (middle) of **Mg-3Et** (A) and **H_2-3Et** (B). On-line MS spectra (lower) of **Mg-3Et** (C) and **H_2-3Et** (D)

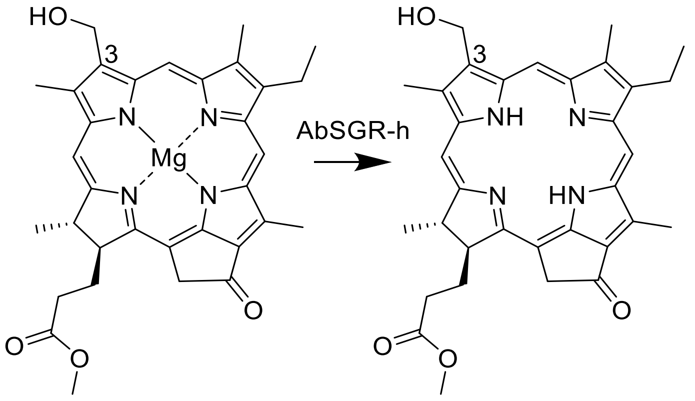

Mg-3HM **H_2-3HM**

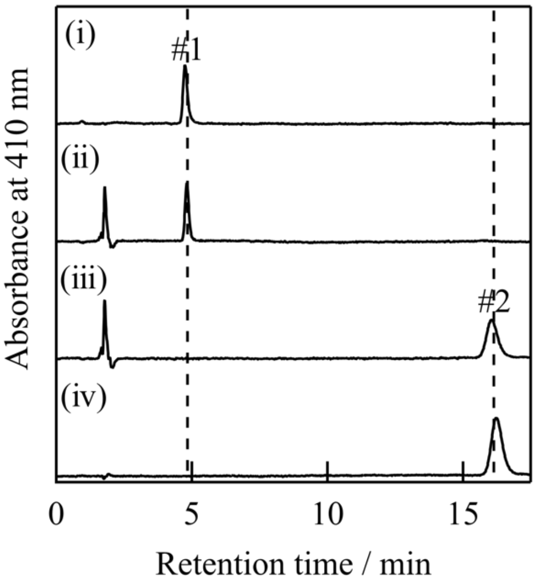

Fig. S7 AbSGR-h activity with **Mg-3HM**. From top to bottom, the HPLC profiles show C3-hydroxymethylated **Mg-3HM** before (i) and after incubation without (ii) and with AbSGR-h for 1 h (iii) as well as authentic product **H_2-3HM** (iv): Cosmosil 5C_{18}-AR-II, 4.6 ϕ × 150 mm; MeOH/H_2O = 85/15 (v/v); 1.0 mL/min. The enzymatic reaction is shown in the activity assay of AbSGR-h *in vitro* in Materials and methods section.

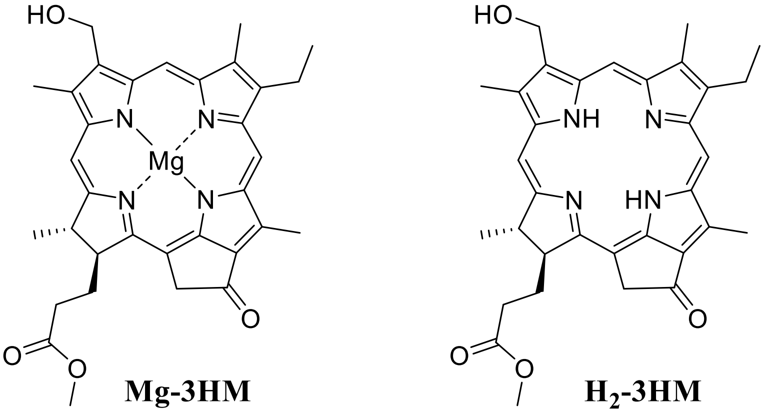

Calcd for $C_{33}H_{35}N_4O_4Mg^+$; 575.2503 Calcd for $C_{33}H_{37}N_4O_4^+$; 553.2809

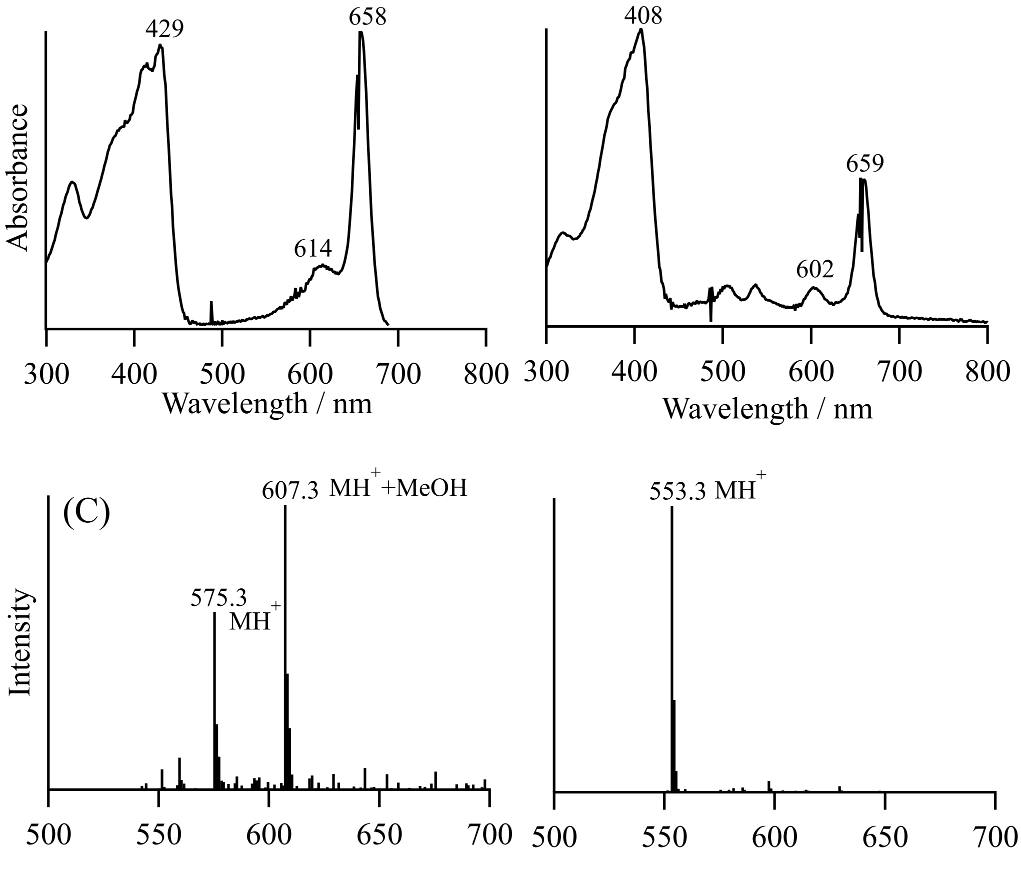

Fig. S8 Chemical structures (upper) of **Mg-3HM** (left) and **H₂-3HM** (right). On-line UV-vis spectra (middle) of **Mg-3HM** (A) and **H_2-3HM** (B). On-line MS spectra (lower) of **Mg-3HM** (C) and **H_2-3HM** (D)

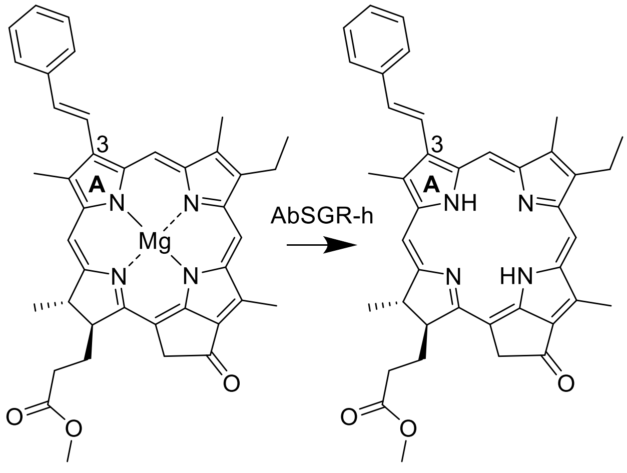

Mg-3TS **H_2-3TS**

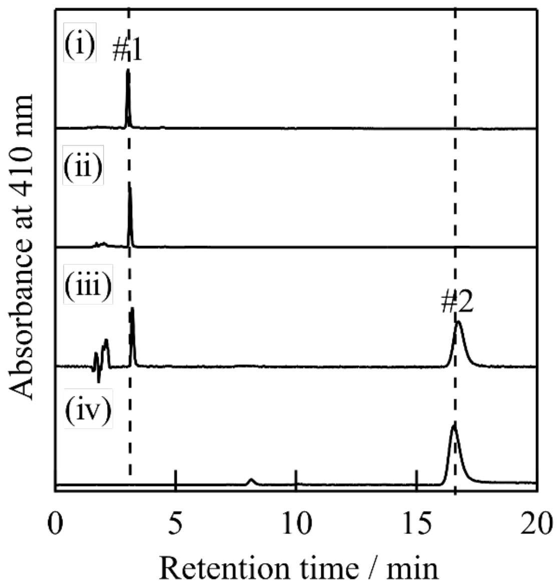

Fig. S9 AbSGR-h activity with **Mg-3TS**. From top to bottom, the HPLC profiles show C3-*trans*-styrylated **Mg-3TS** before (i) and after incubation without (ii) and with AbSGR-h for 1 h (iii) and authentic product **H_2-3TS** (iv): Cosmosil 5C$_{18}$-AR-II, 4.6 ϕ × 150 mm; MeOH/acetone = 99.8/0.2 (v/v); 1.0 mL/min. The enzymatic reaction is shown in the activity assay of AbSGR-h *in vitro* in the Materials and methods section.

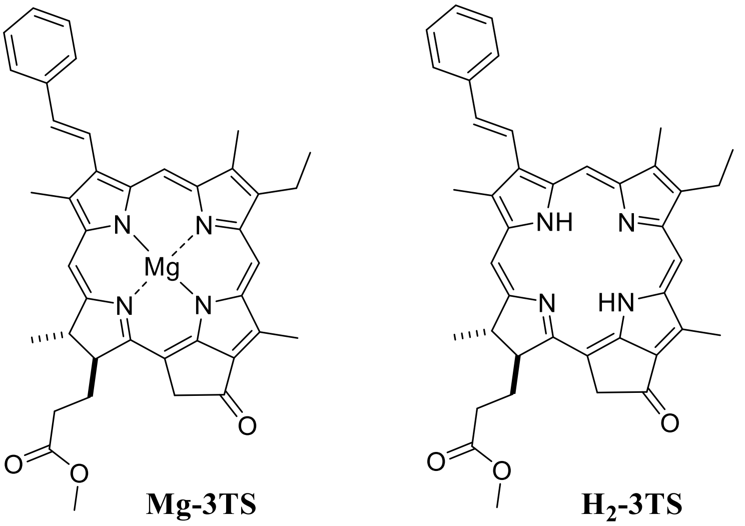

Mg-3TS **H_2-3TS**

Calcd for $C_{40}H_{39}N_4O_3Mg^+$; 647.2867 Calcd for $C_{40}H_{41}N_4O_3^+$; 625.3173

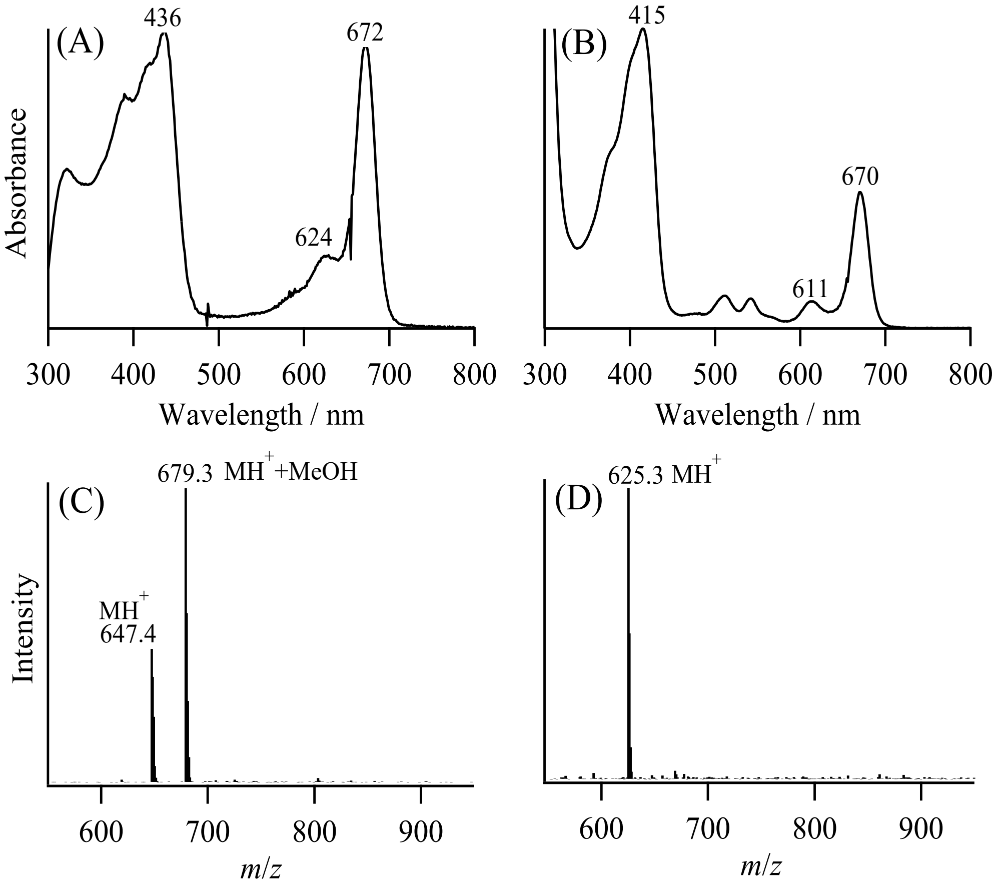

Fig. S10 Chemical structures (upper) of **Mg-3TS** (left) and **H_2-3TS** (right). On-line UV-vis spectra (middle) of **Mg-3TS** (A) and **H_2-3TS** (B). On-line MS spectra (lower) of **Mg-3TS** (C) and **H_2-3TS** (D)

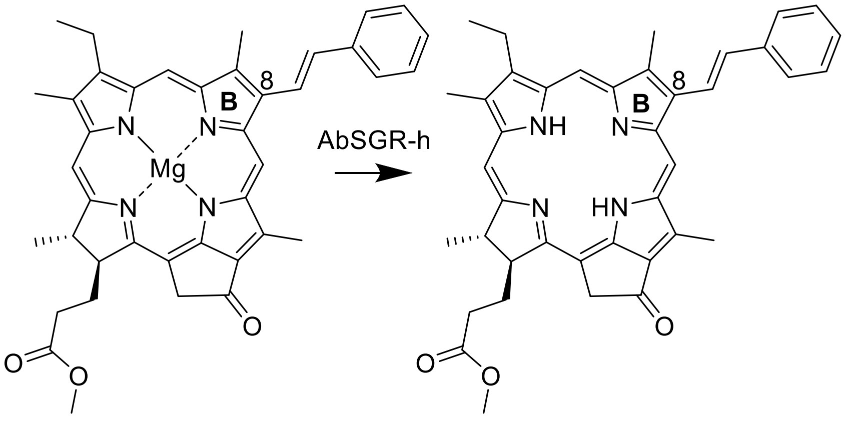

Mg-8TS **H_2-8TS**

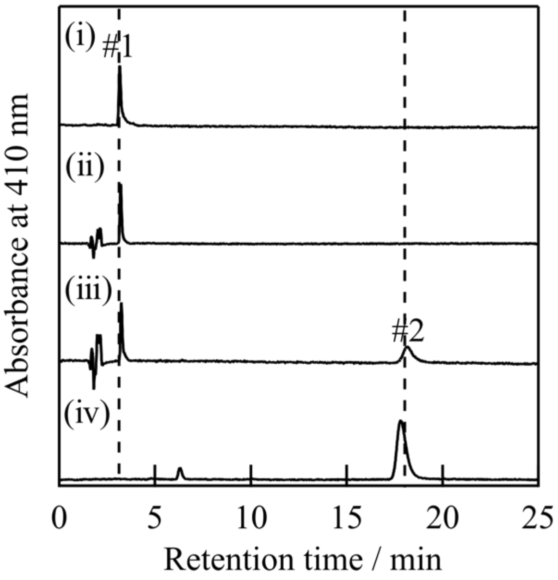

Fig. S11 AbSGR-h activity with **Mg-8TS**. From top to bottom, the HPLC profiles show C8-*trans*-styrylated **Mg-8TS** before (i) and after incubation without (ii) and with AbSGR-h for 1 h (iii) and authentic product **H_2-8TS** (iv): Cosmosil 5C_{18}-AR-II, 4.6 ϕ × 150 mm; MeOH/acetone = 99.8/0.2 (v/v); 1.0 mL/min. The enzymatic reaction is shown in the activity assay of AbSGR-h *in vitro* in the Materials and methods section.

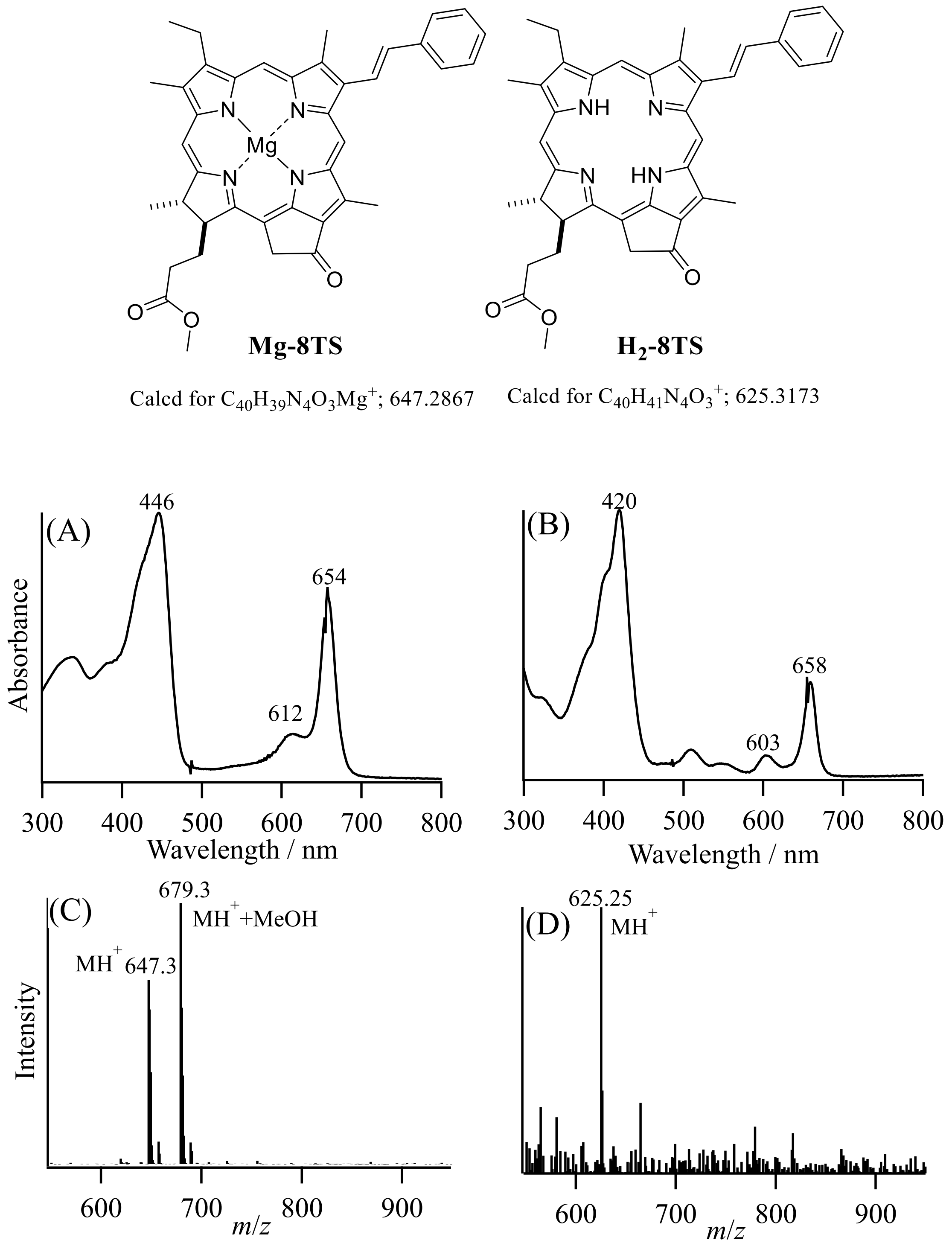

Fig. S12 Chemical structures (upper) of **Mg-8TS** (left) and **H_2-8TS** (right). On-line UV-vis spectra (middle) of **Mg-8TS** (A) and **H_2-8TS** (B). On-line MS spectra (lower) of **Mg-8TS** (C) and **H_2-8TS** (D)

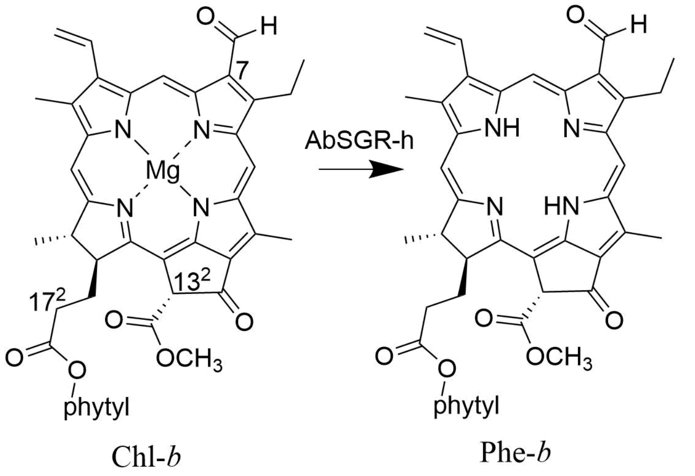

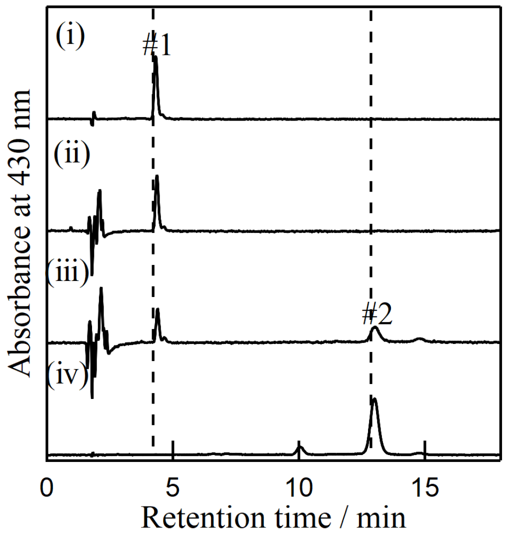

Fig. S13 AbSGR-h activity with Chl-*b*. From top to bottom, the HPLC profiles show Chl-*b* before (i) and after incubation without (ii) and with AbSGR-h for 1 h (iii) as well as authentic product Phe-*b* (iv): Cosmosil $5C_{18}$-AR-II, 4.6 ϕ × 150 mm; MeOH/acetone = 85/15; 1.0 mL/min. The enzymatic reaction is shown in Activity assay of AbSGR-h *in vitro* in Materials and methods section.

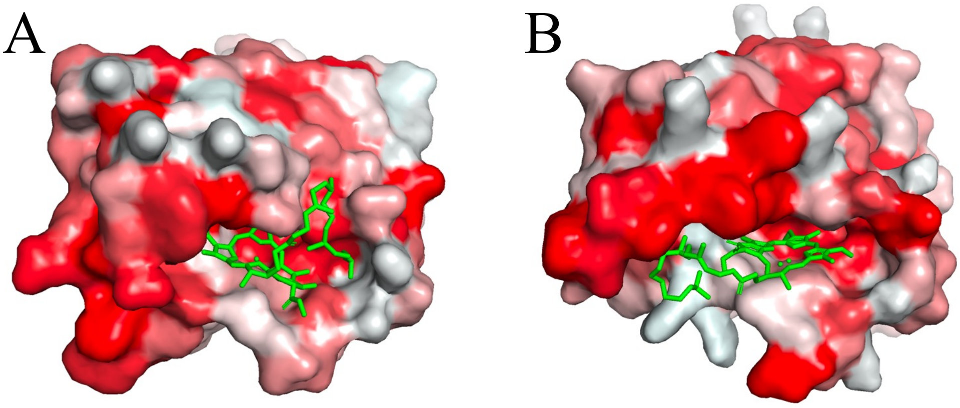

Fig. S14 AbSGR-h (A) and plant SGR docking simulation with Chl-*a* (B). Amino acids are color-coded based on their hydrophobicity, with darker shades of red indicating higher hydrophobicity (Dey et al. 2022).

References

Bible KC, Buytendorp M, Zierath PD, Rinehart KL (1988) Tunichlorin: A nickel chlorin isolated from the Caribbean tunicate *Trididemnum solidum*. Proc Natl Acad Sci USA 85:4582–4586. https://doi.org/10.1073/pnas.85.13.4582

Dey D, Nishijima M, Tanaka R, Kurisu G, Tanaka H, Ito H (2022) Crystal structure and reaction mechanism of a bacterial Mg-dechelatase homolog from the Chloroflexi *Anaerolineae*. Protein Sci 31:e4430. https://doi.org/10.1002/pro.4430

Hirose M, Teramura M, Harada J, Tamiaki H (2020) BciC-catalyzed C13^2-demethoxycarbonylation of metal pheophorbide *a* alkyl esters. ChemBioChem 21:1473–1480. https://doi.org/10.1002/cbic.201900745

Kashiyama Y, Yokoyama A, Kinoshita Y, Shoji S, Miyashiya H, Shiratori T, Suga H, Ishikawa K, Ishikawa A, Inouye I, Ishida K, Fujinuma D, Aoki K, Kobayashi M, Nomoto S, Mizoguchi T, Tamiaki H (2012) Ubiquity and quantitative significance of chlorophyll detoxification catabolism associated with protistan herbivory in aqueous ecosystems. Proc Natl Acad Sci USA 109:17328–17335. https://doi.org/10.1073/pnas.1207347109

Pandey RK, Isaac M, MacDonald Ian, Medforth CJ, Senge MO, Doughterty TJ, Smith KM (1997) Pinacol–Pinacolone rearrangements in *vic*-didehydroxychlorins and bacteriochlorins: Effect of substituents at the peripheral positions. J Org Chem 62:1463–1472. https://doi.org/10.1021/jo960720h

Smith KM, Goff DA, Simpson DJ (1985) *Meso* substitution of chlorophyll derivatives: direct route for transformation of bacteriopheophorbides *d* into bacteriopheophorbides *c*. J Am Chem Soc 107:4946–4954.

https://doi.org/10.1021/ja00303a021

Tamiaki H, Kouraba M (1997) Synthesis of chlorophyll-*a* homologs by Wittig and Knoevenagel reactions with methyl pyropheophorbide-*d*. Tetrahedron 53:10677–10688. https://doi.org/10.1016/S0040-4020(97)00699-6

Tamiaki H, Amakawa M, Shimono Y, Tanikaga R, Holzwarth AR, Schaffner K (1996) Synthetic zinc and magnesium chlorin aggregates as models for supramolecular antenna complexes in chlorosomes of green photosynthetic bacteria. Photochem Photobiol 63:92–99. https://doi.org/10.1111/j.1751-1097.1996.tb02997.x

Tamiaki H, Hamada K, Kunieda M (2008) Synthesis of 3/8-carbonylated chlorophyll derivatives and regiodependent reductivity of their carbonyl substituents. Tetrahedron 64:5721–5727. https://doi.org/10.1016/j.tet.2008.04.025

Tamiaki H, Ariki N, Sugiyama, H, Taira Y, Kinoshita Y, Miyatake T (2013) Synthesis of 3,20-disubstituted chlorophyll-*a* derivatives and reactivity of the substituents. Tetrahedron 69:8412–8421. https://doi.org/10.1016/j.tet.2013.07.060

Tamiaki H, Tsuji K, Kim K, Miyatake T (2016) Preparation of mono-vinylated and formylated chlorophyll derivatives and their optical properties. Tetrahedron 72:4368–4376. https://doi.org/10.1016/j.tet.2016.06.001
